# A Three-Year Biocrime Sanitary Surveillance on Illegally Imported Companion Animals

**DOI:** 10.3390/pathogens10081047

**Published:** 2021-08-18

**Authors:** Monia Cocchi, Patrizia Danesi, Gabrita De Zan, Marta Leati, Laura Gagliazzo, Margherita Ruggeri, Manlio Palei, Alessandro Bremini, Marie-Christin Rossmann, Melanie Lippert-Petscharnig, Michael-Dieter Mansfeld, Silvia Deotto, Sofia Leardini, Federica Gobbo, Paolo Zucca, Paola De Benedictis

**Affiliations:** 1 Istituto Zooprofilattico Sperimentale Delle Venezie, Sezione territoriale di Udine, 33030 Basaldella di Campoformido, Italy; mcocchi@izsvenezie.it (M.C.); gdezan@izsvenezie.it (G.D.Z.); at4ud@izsvenezie.it (S.D.); 2 National Reference Centre/OIE Collaborating Centre for Diseases at the Animal-Human Interface, Istituto Zooprofilattico Sperimentale Delle Venezie, 35020 Legnaro, Italy; pdanesi@izsvenezie.it (P.D.); sleardini@izsvenezie.it (S.L.); fgobbo@izsvenezie.it (F.G.); 3 Laboratory of Parasitology, Istituto Zooprofilattico Sperimentale Delle Venezie, 35020 Legnaro, Italy; 4 National Reference Laboratory for Salmonella, Istituto Zooprofilattico Sperimentale Delle Venezie, Laboratory of Parasitology, 35020 Legnaro, Italy; mleati@izsvenezie.it; 5 Istituto Zooprofilattico Sperimentale Delle Venezie, Epidemiology and Biostatistics, 35020 Legnaro, Italy; lgagliazzo@izsvenezie.it (L.G.); segreteria.scs4@izsvenezie.it (M.R.); 6 Central Directorate for Health, Social Policies and Disabilities, Friuli Venezia Giulia Region, 34123 Trieste, Italy; manlio.palei@regione.fvg.it (M.P.); alessandro.bremini@regione.fvg.it (A.B.); zucca.paolo@regione.fvg.it (P.Z.); 7 Biocrime Veterinary Medical Intelligence Centre, c/o International Police and Custom Cooperation Centre, Thörl-Maglern, 9602 Arnoldstein, Austria; Marie-Christin.Rossmann@ktn.gv.at; 8 Agiculture, Forestry, Rural Areas Veterinary Department, Land Carinthia, 9020 Klagenfurt, Austria; 9 Amt der Kärntner Landesregierung, Institut für Lebensmittelsicherheit, Veterinärmedizin und Umwelt (ILV Kärnten), Laborbereichsleitung Serologie/PCR/Fischdiagnostik, 9020 Klagenfurt, Austria; Melanie.LIPPERT@ktn.gv.at; 10 Amt der Kärntner Landesregierung, Institut für Lebensmittelsicherheit, Veterinärmedizin und Umwelt (ILV Kärnten), Laborbereichsleitung Bakteriologie/Hämatologie, 9020 Klagenfurt, Austria; dieter.mansfeld@ktn.gv.at; 11 Istituto Zooprofilattico Sperimentale Delle Venezie, Laboratory of Special Virology, 35020 Legnaro, Italy; 12 National and FAO Reference Centre for Rabies, Istituto Zooprofilattico Sperimentale Delle Venezie, 35020 Legnaro, Italy

**Keywords:** public health, companion animals, illegal trade, zoonoses, rabies, Canine Parvovirus, Giardia, *Salmonella*

## Abstract

The illegal trade of companion animals in the European Union poses several legal, ethical and health issues to the entire community. In the framework of the Biocrime Interreg project between Italy and Austria, we surveyed puppies and kittens confiscated at the borders to identify the most frequent pathogens associated with (i) the risk of spread within the shelter, (ii) the development of fatal disease and (iii) the zoonotic potential. From January 2018 to December 2020, we examined a total of 613 puppies and 62 kittens coming from 44 requisitions. Feces, skin specimens and blood sera from confiscated animals were tested to verify the presence of major infections and to assess the rabies post-vaccination immunity. Out of the total of individuals under investigation, necropsies and laboratory investigations were also performed on 79 puppies and three kittens that had died during the observation period. Results indicated a high prevalence of Canine Parvovirus (CPV) and Giardia spp. infections, CPV as the most likely cause of fatal gastroenteritis in puppies and *Salmonella* and *Microsporum canis* as major zoonotic pathogens. Conversely, both extended spectrum beta lactamases *Escherichia coli* and methicillin resistant *Staphylococcus pseudintermedius* strains as rare findings. Results highlighted that illegal animal trade could expose the human population to potential zoonotic risk and naïve animal population to potentially disrupting epidemic waves, both of these issues being largely underestimated when buying companion animals.

## 1. Introduction

The illegal trade of companion animals in the European Union poses several legal, ethical and health issues to the entire community. Puppies and kittens are mostly sold online and do not always meet the health requirements established in the European Regulation (EU) 2013/576, being too young to be effectively vaccinated; in addition, they are transported with fraudulent passports which provide false or partial information on their origin [1,2]. Over the years, the Friuli-Venezia Giulia region in Italy and Land Carinthia in Austria have taken up a crucial role as transit routes for the westwards illegal trade rather than being actual regions of destination. For example, in December 2015, the Italian Forestry Corp confiscated a batch of more than 2500 parrots intercepted at the Eastern Italian border [1]. Similarly, illegal routes of pets intended to be sold in Western Europe are randomly discovered in Carinthia.

Based on common experiences and objectives, the Biocrime Interreg Project (www.biocrime.org; accessed on 16 August 2021) was developed between Italy and Austria and funded in 2017 by the European Regional Development Fund Interreg VA Italy Austria. The final aim of the project was to tackle the illegal animal trade through a cross-border cooperation approach, to monitor the health status of traded animals and to protect the health and safety of EU citizens [1]. Indeed, from a sanitary perspective, the illegal animal trade can facilitate the spread across borders of pathogens relevant to both animal and public health [3,4,5,6]. The strategy included the synergic cooperation among veterinary public health, justice and law enforcement through the involvement of the international police and custom cooperation centers [1].

We herein present the results of a three-year surveillance on illegally imported and seized pets. We surveyed puppies and kittens confiscated at the borders for a total of 44 requisitions performed from January 2018 to December 2020. The confiscated animals were subject to a first step screening and to a secondary sampling in case of development of symptoms leading to death while quarantined. Laboratory investigations were intended to rule out the presence of pathogens that might have an impact in terms of public or animal health as associated with wide spreading capacity, high fatality rate and/or zoonotic potential.

## 2. Materials and Methods

### 2.1. Animals and Samples

We surveyed seized puppies and kittens entering the Italian far Eastern border over a three-year period (2018–2020). After arriving at the shelters and according to the capacity of the hosting facility, the quarantined animals were divided into small groups and properly confined in separate enclosures, isolating the groups of one same transport. Usually, within 48 h after arrival a standardized sampling procedure was performed. Briefly, the following samples were collected: blood, skin swabs from auricular, interdigital and abdominal sites, fur and skin material collected through Mackenzie brush technique and feces collected from each individual enclosure. This was decided in order to avoid any individual handling that might have caused distress. Each fecal sample was then pooled with those from the same enclosure (mainly containing ten individuals). In case of critical clinical conditions and dehydration, feces only were collected to minimize animal distress.

Necropsies and laboratory investigations were performed on individuals that had died during the survey. The same standardized sampling protocol including bacteriological, virological and mycological investigations was applied according to macroscopic lesions.

All acronyms used along the text have been listed in Table A1 to allow fluent reading.

### 2.2. Laboratory Testing

All the analyses were performed at the Istituto Zooprofilattico Sperimentale delle Venezie in accordance with ISO 17025, as described in the Quality Manual-General Part, section n° 1. The laboratory is certified by “Accredia”, the Italian national accreditation body. The accreditation code is 0155 N (http://www.accredia.it/en; accessed on 16 August 2021).

#### 2.2.1. Bacteriology

Superficial skin swabs were cultured on blood agar plates and aerobically incubated. Suspected *Staphylococcus* (*S.*) spp. isolates were randomly selected and biochemically confirmed via ID 32 Staph (API^®^, BioMerieux); *S. pseudintermedius* methicillin resistance (MRSP) was performed on selected colonies by disk diffusion test according to the guidelines of the Clinical and Laboratory Standards Institute (CLSI) [7].

Feces were submitted to routine aerobic and anaerobic culture. After incubation, up to five colonies morphologically referred to as *Escherichia (E.) coli* species, were selected and biochemically identified using routine test. The detection of the extended-spectrum-beta-lactamase (ESBL) was performed in accordance with CLSI guidelines [7].

The isolation and identification of *Clostridioides difficile* [8] and *Salmonella* [9], was performed as reported in the literature. For *Salmonella* isolation, in addition to feces, visceral samples were also analyzed during the post-mortem examination. 

*Salmonella* isolates were subjected to phenotypic and molecular characterization. Serotyping was performed according to the ISO/TR 6579-3:2014 method and the *serovar* name was attributed according to the Kauffmann–White–Le Minor scheme. The strains that resulted to be Enteritidis and Typhimurium (monophasic variant) were further characterized using Multilocus variable-number tandem repeat analysis (MLVA) technique [10,11]. MLVA results are reported for both serovars as a string of five numbers representing the number of tandem repeats at the corresponding loci, respectively: SENTR7, SENTR5, SENTR6, SENTR4 e SE3 for *S.* Enteritidis and STTR9– STTR5–STTR6–STTR10–STTR3, for monophasic *Salmonella* Typhimurium; in the event a polymerase chain reaction (PCR) product was not obtained the value “NA” is assigned.

#### 2.2.2. Mycology

Specimens for mycological investigations consisted in fur and skin materials individually collected through the Mackenzie brush technique, preserved in a clean plastic envelope and submitted to the Parasitology Laboratory for culture investigations. Mycological cultures were performed on mycobiotic agar (the mycobiotic agar is produced at the IZSVe laboratory according standardized and quality control procedures). Cultures were considered negative if no colony suggestive to dermatophyte was seen after a 10-day incubation period at 25 °C. Dermatophyte micro-morphology of the colonies was confirmed by assessing lactophenol cotton blue preparations under a light microscope. DNA was then extracted from a single colony and PCR targeting the internal transcribed spacer 1/2 (ITS1/2) regions of the rDNA region was performed [12]. Sanger sequences (600–650 bp) were compared to those publicly available through the Basic Local Alignment Search Tool (BLAST https://blast.ncbi.nlm.nih.gov/Blast.cgi, accessed on 16 August 2021) for appropriate species identification.

#### 2.2.3. Parasitology

Stool samples were tested as follows:

Copromicroscopic technique was performed on 2 g of feces as previously described [13]. Presence of helminths eggs and coccidia oocysts were described according morphological keys [14,15].

One (1) g was submitted to direct immunofluorescent assay for the detection of Cryptosporidium oocyst and Giardia cysts by using the commercial kit MERIFLUOR Cryptosporidium/Giardia^®^ (Meridian Diagnostic, Cincinnati, OH, USA) according to the manufacturer’s instructions.

#### 2.2.4. Virological Investigations

Virological investigations were carried out from pooled fecal samples (intra vitam) or target organs, such as the intestine, liver, lung or central nervous system, collected during necropsies (post mortem). They included quantitative molecular testing for Canine Parvovirus and Feline Panleukopenia [16], Canine Distemper virus [17], Canine Coronavirus, Minute Virus of Canine and Canine Adenovirus 1 and 2 [18]. Briefly, DNA and RNA extraction was performed using QIAsymphony DSP Virus/Pathogen Midi kit (QIAGEN, Hilden, Germany) on the QIAsymphony SP instrument.

The amplification kit QuantiFast^®^ Pathoghen PCR+IC (QIAGEN, Hilden, Germany) was used for the detection of Minute Virus of Canine, Canine Parvovirus, Feline Panleukopenia virus and Canine Adenovirus 1 and 2, while CFX 96 BIO-RAD (BIO-RAD, Hercules, CA, USA.) was used as platform. The amplification kit QuantiTect^®^ Multiplex RT-PCR kit (QIAGEN, Hilden, Germany) was used for the detection of Canine Coronavirus and Distemper virus using respectively the CFX 96 BIO-RAD (BIO-RAD, Hercules, CA, USA.) and Corbett Research Rotor-Gene™ (Corbett Research, Mortlake, Australia) platforms.

#### 2.2.5. Rabies Diagnosis and Rabies Antibody Titration

Rabies infection was ruled out in all the deceased individuals, independently from their symptoms. The central nervous system was tested for the presence of rabies antigen by means of fluorescent antibody test [19] and results were confirmed through rapid tissue culture infection test and a one-step RT-PCR [20].

Rabies post-vaccination immunity was checked through serology using the Fluorescent Antibody Virus Neutralisation test [19]. Before transportation, imported puppies must travel with an international passport certifying their rabies vaccination status [21].

### 2.3. Statistics

Point prevalence of positives over the total of results was calculated with Wilson confidence intervals and 95% probability. When there were no positive results, a unilateral confidence interval was calculated with a lower limit set equal to 0. Significant associations between two different findings on the same sample were calculated using the Fisher non-parametric test, with significance value *p*-value > 0.05. Analyses were performed using R, version 4.1.0.

## 3. Results

### 3.1. Seizures and Sampled Individuals

A total of 675 animals were observed from 44 seizure events. Puppies (*n* = 613) were more frequent than kittens (*n* = 62). The number of intercepted animals increased significantly in 2020 with 414/675 seized individuals (61.33%) versus 110/675 (16.30%) and 151/675 (22.37%) in 2019 and 2018, respectively. Ages ranged from less than two months to over one year. 47.08% were less or equal than three months. The countries of origin of the seized pets mostly belonged to the European Union (i.e., Austria, Croatia, Czech Republic, Hungary, Poland, Romania and Slovakia) (525/675, 77.8%). Non-EU countries of origin included Belarus, Moldova, Serbia and Ukraine (99/675, 14.7%). No information was available on the origin of 51 seized animals (7.6%) (Table S1).

### 3.2. Pathogens Identified from Skin Brush and Skin Swabs

Results from skin swabs performed in dogs (number of swabs = 1413 collected from 613 individuals) showed the presence of *S. pseudintermedius* isolates in 59.5% (CI 57.01–62.12%) of samples (842/1413), while it was an infrequent finding in cats (see Table S2). None of the investigated individuals showed symptoms attributable to *S. pseudintermedius* infection. Only two dogs tested positive to MRSP strains (8/604 replicates). *Microsporum (M.) canis* was the only species of isolated dermatophyte. More in detail, *M. canis* grew in cultures from 5.6% (CI 3.84–8.18%; 25/444) and 6.3% (CI 1.73–20.15%; 2/32) of dogs and cats, respectively (see Table S2).

### 3.3. Pathogens Identified from Pooled Stools

In dog samples, Giardia spp. was the most prevalent enteric protozoa found in 123/256 samples (48.05%, CI 42–54.15%), of which 9 were co-infected with Cryptosporidium spp. Canine Parvovirus (CPV) was identified in 186/227 pools (81.94%, CI 76.41–86.4%), Canine Coronaviruses (CaCoV) in 78/189 pools (41.27%, CI 34.49–48.39%) and Minute Virus of Canine (MVC) in 77/232 pools (33.19%, CI 27.45–39.48%) (Table 1 and Tables S3 and S4). Despite *E. coli* and *C. perfringens* were routinely observed, only one dog sample was identified as hosting an ESBL strain (5/422 replicates) (Table 1). Results obtained from pooled stools collected from seized cats are available as Table S2.

### 3.4. Pathogens Identified from Animals Dead during the Observation Period

Seventy-nine puppies that had died during the observation period were further subjected to necropsy and laboratory analysis to determine the most likely cause of death. Rabies infection was ruled out in all individuals, despite no neurological signs were referred and necropsies showed that signs of gastroenteritis were the most frequent gross findings (Figure S1).

In dogs, CPV was the most frequently identified pathogen, with 67/70 positive intestines (95.71%, CI 88.14–98.53%), followed by MVC (12/72, 16.67%, CI 9.80–26.91%) and CaCoV, with 8/68 positive intestines (11.76%, CI 6.08–21.54%). Giardia spp. was also identified in fecal samples collected post mortem (6/69, 8.70%, CI 4.05–17.70%) (Table 2).

Only 3 kittens were submitted for post mortem investigation (Table S2).

### 3.5. Rabies Post-Vaccination Assessment (Antibody Titration)

**Figure 1:**
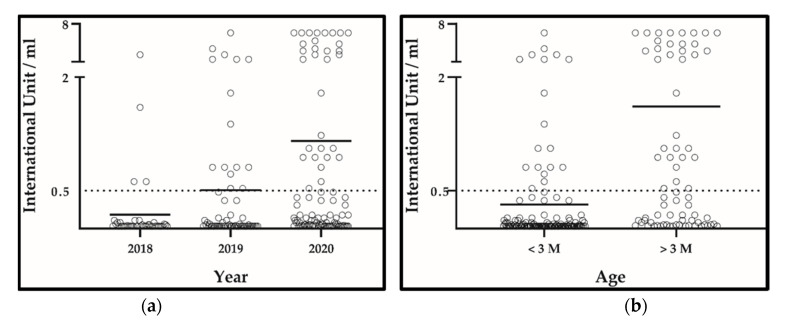
Results and grand mean of rabies antibody titration obtained from 205 puppies. Values are expressed as IU/mL. Values lower than 0.5 IU/mL are classified as negative results. (**a**) Results grouped according to the year of collection (n2018 = 42; n2019 = 65; n2020 = 98). (**b**) Results grouped according to the age category [n1 (<3 months) =133; n2 (>3 months) = 72].

Overall and expectedly, a high rate of rabies vaccination failure was detected in puppies under investigation, with only 51 protected out of 205 individuals under examination (24.88%, CI 19.46–31.22%). Of note, vaccination failure was found in puppies under 3 months (115/133, 86.46%, CI 79.45–91.77%) rather than in the older ones (39/72, 54.16%, CI 42.00–65.97%), with an average value of 0.08 IU/mL (standard deviation 0.101) among negative results, a value well below 0.5 IU/mL the minimum protective standard value following vaccination (Figure 1). 

### 3.6. Salmonella Isolation and Characterization

We were able to isolate 17 *Salmonella* strains out of 250 samples under investigation (6.80%, CI 4.29–10.62%). Of note, cat samples scored positive in 6/24 cases (25%, CI 11.99–44.90%), while dog positive samples were only 11/226 (4.87%, CI 2.74–8.50%). Of note, positive findings were collected mostly from living animals (13/17). Further information on the identification and MLVA characterization are provided in Table 3.

## 4. Discussion

We herewith describe the results obtained from a three-year sanitary surveillance of illegally imported puppies (*n* = 613) and to a lesser extent kittens (*n* = 62) confiscated at the Italian far eastern border. Of note, we testified the occurrence of pathogens either with a paramount impact on animal health or with zoonotic potential. In this latter case, the close contact with companion animals could represent a risk for exchanging pathogens mainly transmitted through the oral-fecal route as well as harbored at skin level. Indeed, healthy individuals represent a high risk as they might silently carry and transmit pathogens to humans, through cohabitation and close relationships with their owners.

Among the pathogens with zoonotic potential at the intestinal level, we found a high prevalence of *Giardia* spp. in pooled stools collected from puppies. Although Giardia is able to infect a wild range of mammals, including humans, its role as zoonotic pathogen is still a matter of discussion [22]. Of note, among the eight assemblages described among the Giardia group, only assemblages A and B are considered infectious for human beings [22]. In this study, no molecular characterization was performed on Giardia cysts, making any isolate potentially zoonotic. The authors therefore suggest that environmental prophylaxis should be applied to prevent host (animal and human) infestation. Indeed, the environmental contamination represents the highest risk of infection not only for Giardia, whose cysts are immediately infectious when shed by the host in the environment [23,24], but also for helminth parasites, such as *Toxocara canis* found in this study.

We isolated 17 *Salmonella* strains and found a higher prevalence in kittens than in puppies and, overall, in healthy individuals. *Salmonella* is a Gram-negative zoonotic bacterium belonging to the family *Enterobacteriaceae*; it is responsible for several cases of human illness worldwide and poses a serious concern in the European Union. Among the *Salmonella* serovars identified in this study, *S.* Enteritidis, the monophasic *S.* Typhimurium, *S.* Infantis and *S.* Hadar are all listed among the 20 serovars most frequently associated with human salmonellosis in Europe [25]. In particular, *S.* Enteritidis and monophasic *S.* Typhimurium are ranked respectively first and third in the list, contributing to almost 60% of all the human cases in 2019 and, thus, their detection deserves greater attention [25]. 

According to the Italian available data (http://entervet.izsvenezie.it, accessed on 16 August 2021), *S.* Enteritidis with MLVA profile 3-9-4-4-1 identified in some of our strains has never been identified in Italy so far. True is that the authors acknowledge that most of the available data refer to isolates collected from farmed species. Indeed, *S*. Enteritidis with MLVA profile 3-9-5-4-1, potentially epidemiologically correlated to 3-9-4-4-1, was reported to have been isolated from humans in 2017 [26], as well as the MLVA profile 2-10-7-3-2, identified for all the strains isolated both from cats and humans in the same year [26], thus suggesting a common source of infection for humans and animals. *S.* Enteritidis with MLVA profiles 2-11-7-3-2, identical to those identified in one of the puppy was identified in human isolates during an outbreak in 2014 [26]. The Italian Enter-Vet database has never notified the presence of monophasic *S*. Typhimurium with MLVA profile 3-12-10-NA-0211; however, it was identified in human isolates in 2010 in England and Wales [27]. Altogether, our data confirm that companion animals could represent a vehicle for *S*. Enteritidis infection to humans, thus representing an underestimated potential human health issue that deserves to be further investigated. Currently, the lack of information on the actual epidemiology of *Salmonella* makes it difficult to define whether its screening should be included in the future legislation for pet movements. Nevertheless, the authors recommend that *Salmonella* should be tested before puppies/kittens are further entrusted to a new owner.

Antimicrobial resistant microorganisms pose a severe threat both to human and animal health, due to the increasing trend of the untreatable bacterial infections and to the reduction of the treatment’s efficacy [28,29]. Bacterial resistance to antimicrobials could occur in food-producing animals, being transmitted to humans via food-borne routes, but also through direct animal contact [29]. In our three-year survey, we found a negligible, low prevalence of puppies carrying ESBL *E. coli* at intestinal level and MRSP at skin level. ESBL-producing bacteria were first identified as nosocomial pathogens of humans, but recently they have appeared also in the community, having a worldwide distribution. ESBL-producing *E. coli* bacteria are described in farm animals, even if an increasing proportion of ESBL has also been reported in *Enterobacteriaceae* isolated from companion animals, where the overall prevalence of ESBL isolates was 2.5% [30]. *S. pseudintermedius* is considered one of the major pathogens in dogs, causing otitis, dermatitis, urinary tract infections and postoperative infections [31]. In addition, this bacterium is also part of the normal flora of healthy dogs [32,33]. Even if the authors assume that its zoonotic potential is not as remarkable as the one observed for *S. aureus*, recent studies have also associated *S. pseudintermedius* to severe bacterial infections in humans [34]. Moreover, methicillin-resistant *S. pseudintermedius* (MRSP) is also emerging in Europe [35]. The knowledge about MRSP carrier prevalence among healthy dogs is limited and studies show that the prevalence of MRSP carriage in healthy dogs may vary from none to 4.6% [36]. MRSP is a matter of growing concern in small animal pathology, having spread quickly since 2005 [35]. Apparently, healthy asymptomatic carriers of MRSP may act as reservoirs and contribute to the spread of the strain to dogs and eventually to humans [37,38]. 

Among zoonotic pathogens transmitted through direct skin contact, we found *Microsporum canis* in 5.6% and 6.3% of healthy puppies and kittens. Although such a prevalence is expected in kittens that are indeed considered as potential reservoirs of such a dermatophyte, the findings from our survey underline the importance of testing asymptomatic puppies as well, similarly to what advised for *Salmonella*.

Rabies was taken into account as well, as an OIE/EU notifiable disease and for the high sanitary impact that such an infection could have if introduced into a rabies-free member state [5,39], although such a risk in Western Europe remains low [40]. Of note, rabies has recently been prioritized among the other high ranking pathogens of possible introduction into the EU [4]. All puppies in our survey were considered as potentially rabies infected and laboratory diagnosis was ruled out in all the confiscated animals. Nevertheless, we found no rabies cases in the animals under investigation. Indeed, the risk of rabies introduction through illegally imported animals seems to be negligible compared to the threat posed by the rescue of stray dogs [39,41]. In fact, the dogs included in our survey were all breeding puppies likely raised in a confined environment and transported immediately after weaning, with poor or no opportunity to acquire rabies infection. On the other hand, we noticed a high rate of rabies vaccination failure, data even worse than the ones reported by previous studies on imported dogs [41,42,43]. As extensively discussed elsewhere, possible explanations for the low rate of rabies neutralizing antibody could be either transport-related stress [41,44] or counterfeit vaccine certificates coupled with a suboptimal age at vaccination [45]. Indeed, we observed a higher vaccination failure in puppies < 3 months.

In our survey, rabies vaccination failure can be considered as a proxy of a more generalized trend of poor health in the analyzed puppies. Indeed, clinical signs and post-mortem lesions observed during our survey were in most cases referable to gastrointestinal involvement frequently associated with CPV infection. Despite the widespread availability of vaccines, such a highly contagious pathogen remains one of the most frequent causes of fatal gastroenteritis in puppies. In this regard, the age of the puppy at administration of the CPV vaccine is considered a significant risk factor for vaccination failure, with a recommended final age at vaccination not younger than six weeks and up to sixteen/twenty weeks for all CPV vaccines [46]. Of note, the puppies in our study were mostly younger than the recommended vaccination age and they were likely exposed to the infection during transportation. Indeed, illegal transport protocols escape the European and national legislations in terms of both animal welfare and sanitary requirements, with the possibility that animals collected from different breeding farms (thus, representing different epidemiological units) are grouped together and travel in suboptimal conditions. The close cohabitation of transported puppies that are too young to be correctly immunized might explain the high CPV prevalence observed in our survey. In addition to representing a paramount issue for puppies, the high rate of CPV infection observed in our survey might also represent an issue for preserving endangered carnivore species in Europe. Of note, pathogens that can be transmitted among multiple host species pose challenges for disease control [47]. Indeed, the interface between wildlife and domestic animals might allow the transmission of pathogens with a potentially disrupting impact on a naïve population [48,49,50,51,52,53]. This occurrence, so far underestimated, deserves further investigations from both sides of the interface.

## 5. Conclusions

Overall, the results of our survey indicate that the number of seized animals was higher in 2020 compared to previous years, which could be explained by several factors. Monitoring and containment actions taken by national and international authorities do not seem to represent a deterrent to fraudulent trafficking, given the economic interests that generate the illegal trade of puppies. The current COVID-19 pandemic may have produced a surge in demand for puppies, mostly requested to ease the psychological distress associated with the COVID-19 lockdown measures applied in most EU countries. The role of pets to the physical and mental well-being of their owners is well known. In addition, the strong uptake of e-commerce has greatly increased online purchases and this has reduced physical retail. The development of the online trade has led to an increase in the pet trade in general and of the illegal trade in particular. Through the direct-to-consumer policy, puppies are shipped from the producers/international distributors to buyers without relying on traditional stores or other middlemen [54]. Although the main targets of illegal pet trafficking are families and adolescents, a high proportion of adolescents does not know that most infectious diseases affecting humans come from an animal reservoir [6]. Therefore, the main target of illegal trafficking is even more exposed to zoonotic risk as largely ignorant. Nevertheless, potential pet owners must be aware that obtaining their pets from the black market might put them at risk of exposure to zoonotic agents and jeopardize the animal’s welfare due to transport-related stress. Highly contagious pathogens might lead to severe, often fatal infectious diseases for both animals and humans that might further spread once introduced into both domestic and wild naive populations with several impacts on both public and animal health as well as wildlife conservation.

## Figures and Tables

**Table 1:** Results from the analyses performed on pooled stools collected from puppies.

Target	Positive/Analysed	Prevalence% (CI%)
*Giardia* spp.	123/256	48.05 (42.00–54.15)
*Cryptosporidium* spp.	9/250	3.60 (1.91–6.70)
*Salmonella*	11/226	4.87 (2.74–8.50)
Canine Parvovirus	186/227	81.94 (76.41–86.40)
Canine Coronavirus	78/189	41.27 (34.49–48.39)
Minute Virus of Canine	77/232	33.19 (27.45–39.48)
Canine Adenovirus type 1	24/188	12.77 (8.73–18.29)
Canine Adenovirus type 2	10/153	6.54 (3.59–11.61)

**Table 2:** Results from analyses performed post-mortem on 79 dog carcasses.

Target	Positive/Analysed	Prevalence% (CI%)
*Giardia* spp.	6/69	8.70 (4.05–17.70)
*Cryptosporidium* spp.	0/69	0.00 (0.00–3.77)
*Salmonella*	4/75	5.33 (2.09–12.92)
Canine Parvovirus	68/73	93.15 (84.95–97.04)
Canine Coronavirus	9/71	12.68 (6.81–22.37)
Minute Virus of Canine	13/75	17.33 (10.42–27.43)
Canine Adenovirus type 1	6/74	8.11 (3.46–16.89)
Canine Adenovirus type 2	1/74	1.35 (0.00–7.27)

**Table 3:** Characterization of the *Salmonella* strains isolated from puppies and kittens.

Year	Origin	Host Species	Characterisation	MLVA Profile
2018	Feaces	Dog	*S.* Infantis	n.p.
2018	Feaces	Dog	*S.* Infantis	n.p.
2018	Feaces	Cat	*S.* Enteritidis	2-10-7-3-2
2018	Feaces	Cat	monophasic *S.* Typhimurium	3-12-10-NA-0211
2020	Feaces	Dog	*S.* Enteritidis	2-11-7-3-2
2020	Intestines	Dog	*S.* Enteritidis	3-9-4-4-1
2020	Intestines	Dog	*S.* Enteritidis	3-9-4-4-1
2020	Intestines	Dog	*S.* Enteritidis	3-9-4-4-1
2019	Feaces	Cat	monophasic *S.* Typhimurium	3-12-10-NA-0211
2019	Feaces	Cat	*S.* Enteritidis	2-10-7-3-2
2019	Feaces	Cat	*S.* Enteritidis	2-10-7-3-2
2019	Feaces	Cat	*S.* Enteritidis	2-10-7-3-2
2020	Feaces	Dog	*S.* Corvallis	n.p.
2020	Feaces	Dog	*S.* Debou	n.p.
2020	Feaces	Dog	*S.* Hadar	n.p.
2020	Feaces	Dog	*S.* Bredeney	n.p.

n.p. not performed. MLVA profile characterization was performed on *S.* Enteritidis and monophasic *S.* Thyphimurium only.

## Data Availability

Not applicable.

## Supplementary Materials

The following are available online at https://www.mdpi.com/article/10.3390/pathogens10081047/s1, Table S1: Origin of seized companion animals per year (2018–2020). Table S2: Laboratory results from seized kittens (2018–2020). Table S3: Distribution of results (CPV Vs. CaCoV) on pooled stools from puppies (2018–2020). Table S4: Distribution of results (CPV Vs. Giardia) on pooled stools from puppies (2018–2020). Figure S1: Puppies’ small intestines from dead individuals.

## Appendix A

**Table A1:** Glossary of the acronyms used along the text. Acronyms are listed in alphabetical order.

Acronym	Meaning
CaCoV	Canine Coronavirus
CI	Confidence Interval
CLSI	Clinical and Laboratory Standard Institute
COVID-19	COronaVIrus Disease 2019
CPV	Canine Parvovirus
ESBL	Extended-spectrum-beta-lactamase
EU	European Union
IU/mL	International Unit/mL
MLVA	Multilocus variable-tandem repeat analysis
MRSP	Methicillin restistant *Staphilococcus pseudintermedius*
MVC	Minute Virus of Canine
