# Supplementary material for: A Three-Year Biocrime Sanitary Surveillance on Illegally Imported Companion Animals
Source: Pathogens. 2021 Aug 18;10(8):1047. doi: 10.3390/pathogens10081047 (PMC8399716; doi:10.3390/pathogens10081047)

Table S1. Origin of seized companion animals per year (2018-2020).

Year	EU membership	Country	Puppies	Kittens	Total
2018	MS	Austria	21		21
		Czech Republic	6		6
		Hungary	56		56
		Romania	10	4	14
		Slovakia	9		9
	Non MS	Serbia	4		4
		Ukraine		35	35
	No data	No data	6		6
2019	MS	Austria	27		27
		Hungary	32		32
		Slovakia	29	4	33
	Non MS	Serbia	9		9
		Ukraine		5	5
	No data	No data	4		4
2020	MS	Croatia	8		8
		Hungary	153	1	154
		Poland	5		5
		Romania	19		19
		Slovakia	141		141
	Non MS	Belarus	14		14
		Moldova	7	6	13
		Serbia	12		12
		Ukraine		7	7
	No data	No data	41		41
		Total	613	62	675

Table S2. Laboratory results from seized kittens (2018-2020).

Target	Positive/analyzed	Prevalence % (CI%)
Giardia spp.	0/42	0.00 – 8.38 (*)
Cryptosporidium spp.	0/42	0.00 – 8.38 (*)
Salmonella	6/24	11.99 – 44.89
Feline Panleukopenia virus	1/12	1.48 – 35.39
Feline Coronavirus	3/12	8.89 – 53.23
S. pseudointermedius	4/66	2.38 – 14.57
Microsporum canis	2/32	1.73 – 20.14

(*) One-sided 97.5% confidence interval

Table S3. Distribution of results (CPV *Vs* CaCoV) on pooled stools from puppies (2018-2020).

**Target**		**Canine Coronavirus**		
		NEGATIVE	POSITIVE	TOTAL
**Canine Parvovirus**	NEGATIVE	15<br>*55.6%*	12<br>*44.4%*	27<br>*100.0%*
	POSITIVE	88<br>*58.3%*	63<br>*41.7%*	151<br>*100.0%*
	TOTAL	103<br>*57.9%*	75<br>*42.1%*	178<br>*100.0%*

Table S4. Distribution of results (CPV *Vs* Giardia spp.) on pooled stools from puppies (2018-2020).

**Target**		**Giardia spp.**		
		NEGATIVE	POSITIVE	TOTAL
**Canine Parvovirus**	NEGATIVE	9<br>*31.0%*	20<br>*69.0%*	29<br>*100.0%*
	POSITIVE	39<br>*40.2%*	58<br>*59.8%*	97<br>*100.0%*
	TOTAL	48<br>*38.1%*	78<br>*61.9%*	126<br>*100.0%*

Figure S1. Puppies' small intestines of dead individuals. A: enteritis, acute with serosal fibrinous exudation and hemorrhages. B: enteritis, acute with serosal haemorrhages. C: enteritis, acute, necrotico-haemorragic, diffuse, severe.

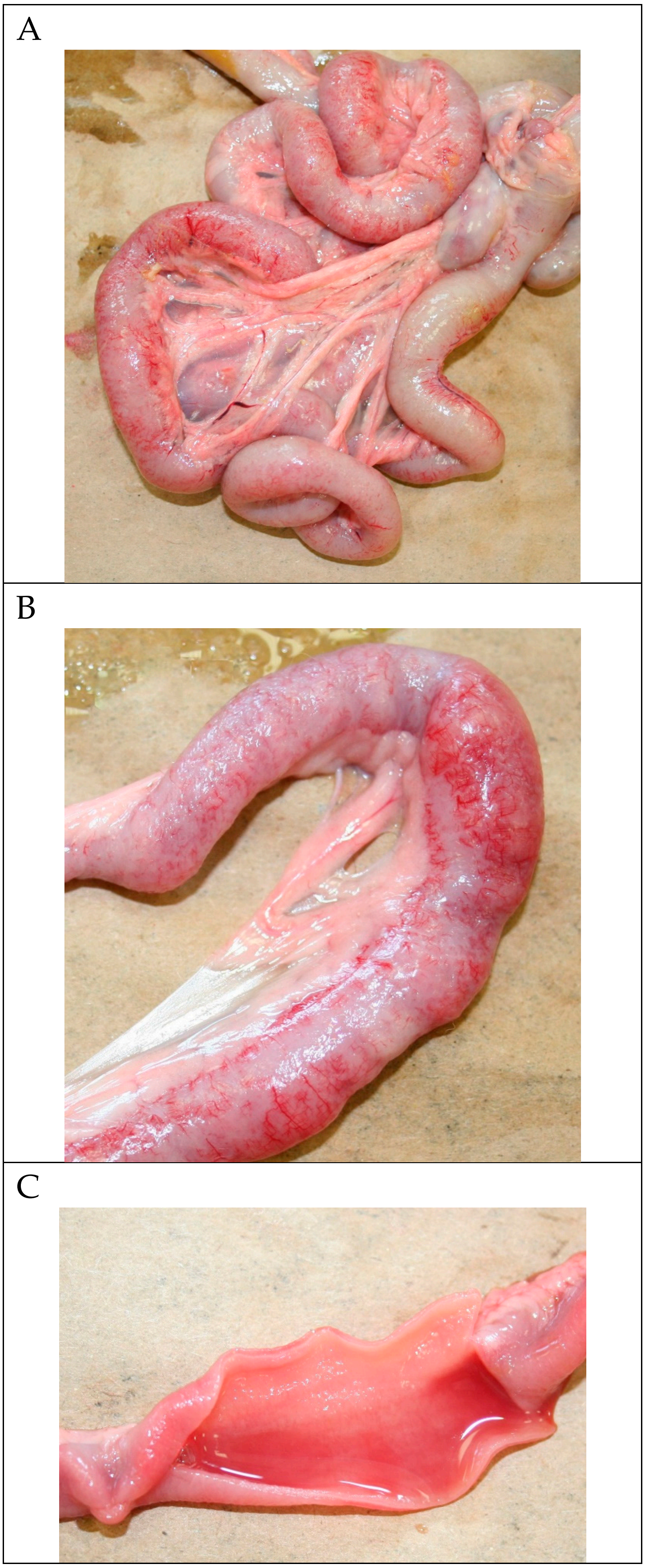

Supplement: Supplementary file 1 [file pathogens-10-01047-s001.zip › pathogens-1320176-supplementary.pdf]
